# Preparation of Fucoxanthin Nanoemulsion Stabilized by Natural Emulsifiers: Fucoidan, Sodium Caseinate, and Gum Arabic

**DOI:** 10.3390/molecules27196713

**Published:** 2022-10-09

**Authors:** Najmeh Oliyaei, Marzieh Moosavi-Nasab, Nader Tanideh

**Affiliations:** 1 Stem Cells Technology Research Center, Shiraz University of Medical Sciences, Shiraz 7193635899, Iran; 2 Seafood Processing Research Center, School of Agriculture, Shiraz University, Shiraz 7144113131, Iran; 3 Department of Food Science and Technology, School of Agriculture, Shiraz University, Shiraz 7144113131, Iran; 4 Department of Pharmacology, School of Medicine, Shiraz University of Medical Sciences, Shiraz 7134853185, Iran

**Keywords:** nanoemulsion, fucoxanthin, fucoidan, natural emulsifiers

## Abstract

This study was proposed to investigate the possibility of O/W nanoemulsion stabilization via natural emulsifiers as a delivery system for fucoxanthin. Nanoemulsions were prepared using ultrasonic treatment (150 W, amplitude 80%, 10 min) with different levels (0.5%, 1%, and 2% wt) of fucoidan, gum Arabic, and sodium caseinate as natural emulsifires and they were compared with tween 80. Then, the creaming index, stability, encapsulation efficacy, Fourier-transform infrared (FT-IR) spectroscopy, and in vitro release were evaluated. The best stability and lowest creaming index were observed at 2% wt of emulsifiers. Nanoemulsions with droplet sizes (113.27–127.50 nm) and zeta potentials (−32.27 to −58.87 mV) were prepared. The droplet size of nanoemulsions was reduced by increasing the emulsifier concentration, and the best nanoemulsion stability after 15 days of storage was in the following order: tween 80 > sodium caseinate > fucoidan > gum Arabic. The encapsulation efficacy of nanoemulsions stabilized by sodium caseinate, fucoidan, and gum Arabic were 88.51 ± 0.11%, 79.32 ± 0.09%, and 60.34 ± 0.13%, respectively. The in vitro gastrointestinal fucoxanthin release of nanoemulsion stabilized with tween 80, sodium caseinate, fucoidan, and gum Arabic were 85.14 ± 0.16%, 76.91 ± 0.34%, 71.41 ± 0.14%, and 68.98 ± 0.36%, respectively. The release of fucoxanthin from nanoemulsions followed Fickian diffusion. The FTIR also confirmed the encapsulation of fucoxanthin.

## 1. Introduction

In recent years, seaweeds and their bioactive compounds have received special attention because of their biological properties as ingredients within the functional food and nutritional industries. Fucoxanthin, the main carotenoid of brown seaweeds and microalgae, is greatly interest as it exhibits considerable in a wide range of healthcare products because of its various biological attributes such as antioxidant activity, anti-obesity, anti-diabetic, anti-cancer, anti-inflammatory, skin-protective, bone-protective, and anti-microbial properties [1].This pigment is found in different algae such as *Laminaria*, *Undaria*, *Sargassum*, *Phaeodactylum*, *Isochrysis*, *Fucus*, *Nitzschia*, and *Chaetoceros* [2]. However, fucoxanthin utilization is limited because its structure contains several conjugated double bonds that make it unstable against light, oxygen, pH, and thermal degradation [3]. In addition, encapsulation is an efficient and promising technique for preserving the bioactive agents from adverse conditions. 

Among various encapsulation techniques, the nanoencapsulation approach is the novel trend in food technology to entrap the hydrophobic bioactive constituents into biopolymers to improve their stability, better controlled delivery or absorbance through gastrointestinal tracts [4,5]. Nanoemulsions are a mix of oil droplets and water, which are stabilized with an emulsifier and have a droplet size ranging from 20 nm to 200 nm. These systems are widely used for encapsulation of different bioactive compounds because of their high stability, good dispersibility, easy production, low opacity, and high surface area [6] and are usually applied in food and pharmaceutical formulations [4,7] to improve the absorption, bioavailability, and delivery of incorporated bioactive compounds [8]. There are several studies using emulsions for fucoxanthin encapsulation by various methods or lipid types [9,10,11,12]. Among the several methods of nanoemulsion preparation, ultrasonication is one of the appropriate approaches because ultrasonic waves create disruptive forces, which break large oil droplets and develop the nanoemulsion. Indeed, during cavitation, the small bubbles within a liquid are formed and rapidly collapsed resulting in creating the nano-size droplets and dispersion [8]. However, emulsions are unstable systems, and their stability can be improved by adding emulsifiers. Emulsifier agents are crucial surface-active ingredients for the formation and stabilization of emulsions by reducing the interface tension and coating the droplets [13]. Several emulsifiers often used in various industries, such as tweens and spans, are synthetic [14]. Tween 80 is an emulsifier often used in various industries, in particular in foods [15]. However, with increasing consumer demand for the healthier products, the utilization of natural food-grade emulsifiers is becoming popular, especially in food products [16]. In recent years, some natural emulsifiers are used in different emulsion systems [13,17,18]. There are various usual types of natural emulsifiers including proteins, polysaccharides, phospholipids, biosurfactants, and bioparticles [14]. Polysaccharides are widely used in food formulations as thickening, gelling, and stabilizing agents. Several polysaccharides, such as seaweed polysaccharides, pectin, and plant-based polysaccharides, are able to act as emulsifiers by enhancing the viscosity and electrostatic repulsion between the droplets [19]. Fucoidan is one of the most abundant seaweed-sulfated polysaccharides in the cell walls of brown seaweeds such as *Fucus*, *Ascophyllum*, *Saccharina*, and *Sargassum* [20]. Fucoidan structure is composed of (1-3)- and (1-4)-linked α-l-fucopyranose units and has sulfate groups at the C-2, C-4, or C-3 [21]. Another polysaccharide that can be used as emulsifier is gum Arabic. This polysaccharide is obtained from exudates of Acacia senegal trees and consists of arabinogalactan, arabinogalactan protein, and glycoprotein. Gum Arabic has a negative charge at pH > 2.2 [17] and is a natural complex with appropriate emulsifying attributes, high water solubility, and low viscosity and is widely used as an encapsulating agent [22].

In addition, the emulsifying capacity of proteins is well known. However, ionic strength, temperature, and pH have an impact on their emulsion-forming properties [23]. Among the protein-based emulsifiers, sodium caseinate is often used as emulsifying agent because of its high solubility, surface activity, thermal resistance, and inhibiting the droplets aggregation by electrostatic and steric repulsion [24,25,26].

Because of consumer demand for using natural and safe ingredients, the present research aims to investigate the natural emulsifiers’ function compared with tween 80 for stabilizing nanoemulsions. Thus, fucoxanthin derived from *Sargassum angustifolium* was encapsulated in an oil in water (O/W) nanoemulsion by the ultrasound technique. Emulsions were prepared by fucoidan, gum Arabic, casein, and tween 80 as emulsifiers and their stability, particle size, zeta potential, encapsulation efficacy, morphology, and in vitro release were investigated. 

## 2. Results

### 2.1. Emulsion Stability and Creaming Index

**Figure 1:**
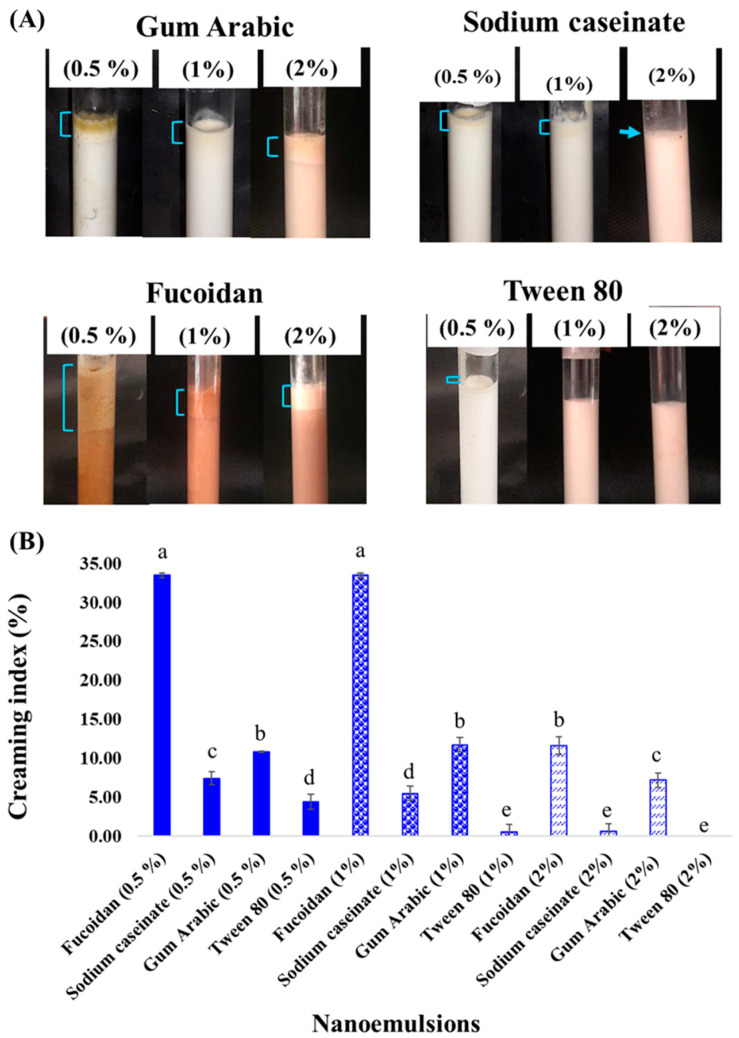
Emulsion stability (**A**) and creaming index (**B**) of different nanoemulsions. Different lowercase letters indicate significant differences (*p* < 0.05).

The emulsion stability and creaming index of different nanoemulsions were evaluated after 15 days (Figure 1). According to the results, the emulsion stability and creaming index decreased at higher emulsifier concentrations (from 0.5 to 2% wt), which can be related to the smaller size of oil droplets. Moreover, by increasing the biopolymer concentration, the negative charge and electrostatic repulsion were enhanced, resulting in an improvement in emulsion stability. Among all natural emulsifiers, sodium caseinate provided higher stability compared with others (*p* < 0.05), while a layer of oil droplets was obtained in the emulsion prepared with 0.5% gum Arabic (Figure 1A). The higher creaming index in samples was due to the lower concentration of emulsifiers. Moreover, no creaming was observed in samples that contained 1 to 2% of tween 80 (Figure 1B), whereas, at the same concentration of the natural emulsifiers, phase separation was detected with a dense cream (*p* > 0.05). In the samples prepared with natural emulsifiers, the creaming index was reduced by increasing the biopolymer concentration, and no significant differences were observed between emulsions stabilized with tween 80 (2%) and those containing 2% sodium caseinate (*p* > 0.05). In this case, the stabilization was ensured by adequate surface-active casein present in the emulsion. The protein types of emulsifiers with HLB values of about 13.5–14 make them appropriate for stabilizing the O/W emulsions [27]. As Liu, Wei [27] reported, gum Arabic and sodium caseinate have HLB 8 and 14, respectively. Tween 80 has an HLB of about 15 [28], which causes higher stability of the emulsion. The substitution of protein (sodium caseinate) with polysaccharides (gum Arabic and fucoidan) altered the emulsion stability. This phenomenon is related to bridging flocculation. A higher rate of creaming was observed in the samples emulsified with carbohydrate, in particular fucoidan (*p* < 0.05). Generally, polysaccharides can impact on the emulsion stability via different approaches including bridging flocculation by binding to the surface of several oil droplets, producing electrostatic repulsion, depletion flocculation, and enhancing the viscosity of emulsion. Bridging flocculation may also take place at lower levels of polysaccharides because of an inadequate amount of polymer for the droplet-coating surface. While the intermediate levels of polysaccharides are enough for surrounding oil droplets and forming stable emulsion [29]. These thick charged layers produce a strong steric and electrostatic repulsion between the oil droplets and prevent aggregation. Although emulsion is stable at high concentration of polysaccharides, depletion flocculation may take place because of non-adsorbed polysaccharides [30]. Furthermore, the molecular properties, charge density, and conformation of sulfated polysaccharides influence their emulsifying ability [29]. The larger structure of fucoidan compared with gum Arabic provides more adsorption sites for droplets resulting in bridging flocculation and promoting several droplet aggregations [17]. Another factor that has an important influence on emulsion stability is droplet size. Generally, the smaller droplet size of <200 nm makes nanoemulsions resistant to physical stability against gravitational separation, droplet aggregations, and Ostwald ripening. Moreover, ultrasonic emulsification is a powerful method for nanoemulsion preparation and shear stress, turbulence, and cavitation disrupt the emulsion into the nanoscale droplet [31]. According to the results, emulsions prepared with 1% and 2% of emulsifiers were selected for further analysis.

### 2.2. Particle Size and PDI

**Figure 2:**
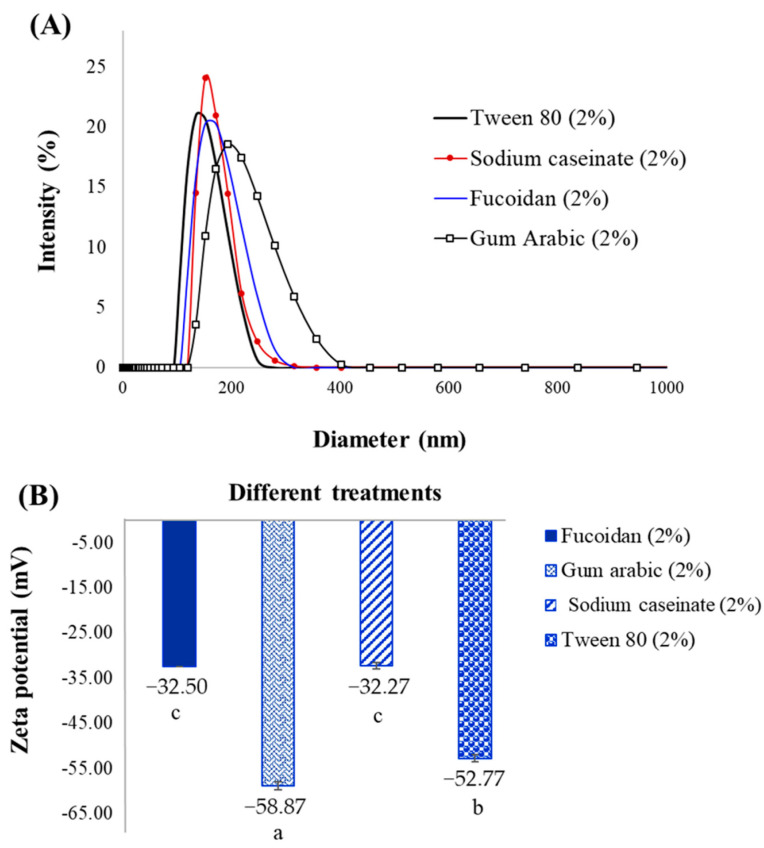
Particle size (after 15 days) (**A**) and zeta potential of different nanoemulsions prepared with 2% emulsifier (**B**). Different lowercase letters indicate significant differences (*p* < 0.05).

From the results (Table 1), the average particle size of nanoemulsions was significantly (*p* < 0.05) influenced by emulsifier type and concentrations. The average size of nanoemulsions prepared with natural emulsifiers was in the range of 161.80 ± 1.95 nm to 143.67 ± 3.35 nm at 1% and decreased to 113.43 ± 1.29–127.50 ± 2.62 nm at 2% of biopolymer concentration. While the droplet size of the sample emulsified with 1% tween 80 was much smaller than the others (*p* < 0.05). Among natural emulsifiers, the casein-stabilized nanoemulsion (2%) possessed a smaller average particle size (113.27 ± 0.45 nm), which had no statistical differences with sample containing 2% tween 80 (*p* > 0.05). These results confirmed that the higher stability of tween 80- and casein-stabilized emulsions was due to the smaller particle size. As depicted in Figure 2A, the droplet size of emulsions (2%) increased after 15 days of storage, and the final droplet size of the emulsions prepared with fucoidan (about 239.73 nm) and gum Arabic (about 262.40 nm) was large than other samples (less than 200 nm). It seems that the nanoemulsions are free from the bigger droplets obtained. These results confirmed the phase separation creaming index of the emulsion after 15 days.

The droplet size was reduced by increasing the emulsifier concentration because there was not an adequate emulsifier to cover the oil droplets at a lower emulsifier concentration. Moreover, different types of emulsifiers had different interfacial behavior, which was attributed to their structures. Indeed, the ability of emulsifiers for reducing surface tension is attributed to their structure. It was observed that casein showed good results of emulsion stabilization because of its more hydrophobic functional groups, which can surround oil droplets faster and produce smaller droplet size, while gum Arabic has a gyration radius of globular configuration (21−28 nm) in its structure [32]. In addition, a larger droplet size enhances the creaming index by increasing the net force acting on the particles resulting in instability of the emulsion. Moreover, emulsifiers with lower molecular weight or with high HLB usually are able to produce smaller droplets in O/W emulsions [33]. A similar observation was reported by Dwyer et al. [34], who investigated the effect of sodium caseinate concentration (0.25% to 3%) on droplet size and stability of O/W emulsion. They claimed that the particle size decreased by increasing the emulsifier concentration. Saravana et al., [35] also reported similar results for fucoxanthin rich oil nanoemulsion stabilized by k-carrageenan. The authors used ultrasound-assisted emulsification for producing nanoemulsion [35]. Our results were in agreement with Ma, Zou [32], who confirmed the effect of type and concentration of natural emulsifiers on the fucoxanthin-encapsulated emulsion. The droplet size of emulsion stabilized by 2 wt% gum Arabic was larger than whey protein isolate (WPI) and modified lecithin. They claimed that the surface area of droplets in gum Arabic-stabilized emulsion was smaller, though it was not as good as WPI to prevent the degradation of fucoxanthin [32]. In addition, the PDI of the nanoemulsion prepared with 2% emulsifiers was in the range of 0.29–0.36 with no significant differences (*p* > 0.05). The relatively narrow particle size distribution in high concentrations of emulsifiers indicated homogeneity in the emulsion.

### 2.3. Zeta Potential

The zeta potential of different samples is shown in Figure 2B. According to the results, all nanoemulsions had a negative charge at neutral pH, and emulsions prepared with gum Arabic (−58.87 ± 0.98 mV) and tween 80 (−52.77 ± 0.75 mV) were extremely negative (*p* < 0.05). However, there were no significant differences between samples containing fucoidan and sodium caseinate (*p* > 0.05). The emulsion stability is highly dependent on the emulsifier’s ability to adsorb at the oil–water interface and the reduction of the surface tension. Proteins are able to enhance the emulsion stability against droplet coalescence via formation the viscoelastic interfacial multilayers [26]. The negative zeta potential of emulsions revealed appropriate physical stability as a result of the strong electrostatic repulsion between particles resulting in preventing the droplet aggregation [29]. Indeed, the high surface charge is responsible for electrostatic repulsions and reducing the van der Waals force between the particles. Usually, agglomeration tends to happen due to the van der Waals forces [36].

However, the polysaccharides-stabilized emulsion had lower stability compared with others. This observation indicated that not only the electrostatic repulsion between the dispersed droplets, but also the structure and molecular weight of the biopolymers, play a significant role in the final stability. Similarly, Gasa-Falcon, Arranz [33] and Ma et al., [32] obtained the negative zeta potential for nanoemulsions stabilized with sodium caseinate and gum Arabic, respectively, which was in agreement with our results.

### 2.4. Encapsulation Efficiency (EE)

Table 2 shows the EE of fucoxanthin present in nanoemulsions. The EE of the emulsions increased significantly (*p* < 0.05) by increasing the emulsifier concentrations from 1% up to 2% for all types of emulsifiers. The highest EE value was obtained with nanoemulsion stabilized with 2% tween 80 (92.15 ± 0.15%). Among natural emulsifiers, nanoemulsion prepared with sodium caseinate (88.51 ± 0.11%) had the highest EE compared with those containing fucoidan (79.32 ± 0.09%) and gum Arabic (60.34 ± 0.13). The type and concentration of wall materials had an influence on emulsion EE. The increase in the amount of biopolymer resulted in higher EE because high amounts of wall material effectively protect and inhibit the oil leakage [37]. The EE results showed an increasing trend when the concentration of emulsifiers was increased. This revealed that oil droplets are adequately coated by emulsifiers and provide an effective encapsulation for fucoxanthin. The high EE values of emulsion stabilized by fucoidan might be related to the precipitation of fucoidan in the presence of ethanol and flocculation resulting in entrapping fucoxanthin in the polysaccharide network. Generally, the obtained results showed that the fucoxanthin was enclosed and entrapped more efficiently in the fucoidan-based emulsion as well as emulsion prepared with sodium caseinate. Similar results were found on fish oil encapsulated in fucoidan-WPI and inoline-WPI with EE values of 86.31% and 81.23%, respectively. The addition of fucoidan resulted in irreversible aggregates that led to droplet coalescence [38]. Fan, Lu [39] also reported the EE of curcumin encapsulated by soy protein isolate–fucoidan nanoparticles was about 96.65%. They also claimed that the curcumin concentration affected EE, and EE was reduced by increasing the curcumin concentration, which led to larger particle size. Li et al., [40] also reported the high EE (>98%) for protein-based encapsulation of fucoxanthin due to its positive influence on carotenoid absorption. They used bovine serum albumin to fabricate with particle size in the range of 113–193 nm. Additionally, the high EE (about 95%) of astaxanthin by bovine serum albumin–fatty acid nanoparticles was reported by Huang et al. [41].

### 2.5. FTIR Spectra

**Figure 3:**
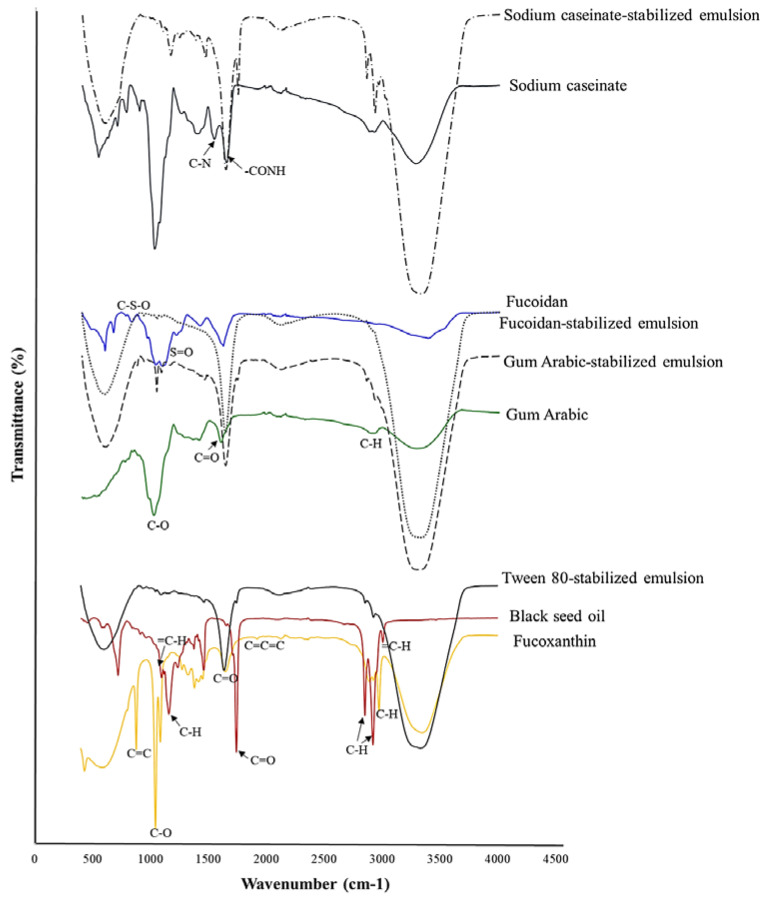
FTIR spectra of fucoxanthin, fucoidan, black seed oil, and their nanoemulsions.

The spectroscopic profiles of fucoxanthin and its emulsions prepared with 2% emulsifier are shown in Figure 3. In FTIR spectra of fucoxanthin, a peak that appears at wavenumbers of 3346 cm^−1^, 2974 and 2895 cm^−1^, and 1940 cm^−1^, caused by stretching O-H groups, alkanes with C-H, and allene (C=C=C), respectively. In addition, the wavenumbers of 1648 cm^−1^ (C=O), 1086 and 1044 cm^−1^ (C-O), and 878 cm^−1^ (C=C) were detected [1]. The spectroscopic profile of the black seed oil is constituted by stretching =C-H alkene (3008 cm^−1^) and C-H in –CH_3_ and –CH_2_ (2922 and 2853 cm^−1^). The peak at 1743 cm^−1^ was attributed to C=O stretching in ketone groups, and 1160 cm^−1^ and 1097 cm^−1^ were assigned to the C-O and =C-H bending groups [42]. All of these groups assessed for the presence of thymoquinone, dithymoquinone, thymohydroquinone, and thymol. A peak with weak intensity at 3008 cm ^-1^ indicates the C-H stretching of the vinyl group [42]. These results were in agreement with Mohammed et al. [42]. In the FTIR spectrum of nanoemulsion stabilized with tween 80, three main peaks at 3332 cm^−1^ (OH), 1637 cm^−1^ (quinoid ring C=O), and 597 cm^−1^ (aromatic ring C-H) were observed [43]. Additionally, numerous bands of black seed oil were disappeared or reduced. The broad peak at about 3332 cm^−1^ indicates the presence of more amounts of OH groups.

In FTIR spectra of fucoidan, the peak at 3383 cm^−1^ was assigned to the OH and H_2_O. The absorbance peaks around wavenumbers of 1037–1213 cm^−1^ and 827 cm^−1^ were caused by stretching vibration of S=O and C-S-O in sulfated groups, respectively. Additionally, the wavenumber at 671 cm^−1^ was identified as deoxysugars such as fucos [44,45,46]. In FTIR spectra of fucoidan-stabilized nanoemulsion compared with fucoxanthin, the main peaks disappeared. Additionally, the sharp peaks of fucoidan and fucoxanthin, especially bands around 1200–1000 cm^−1^ disappeared, and the peak 1618 cm^−1^ shifted to 1635 cm^−1^ and the peak 599 cm^−1^ shifted to 588 cm^−1^; these changes indicate the interaction of fucoidan, black seed oil, and fucoxanthin. Similar FTIR patterns were reported by Zhang et al. [47] for encapsulation of curcumin with fucoidan. FTIR peaks of gum Arabic arose at 3287 cm^−1^ for OH stretching vibration, 2915 cm^−1^ for C-H stretching, 1599 cm^−1^ for C=O stretching, and 1019 cm^−1^ for C-O vibration [22]. After encapsulation of fucoxanthin with the gum Arabic-stabilized nanoemulsion, the high intensity peak appeared at 3315 cm^−1^, which was assigned to OH. It seems that the bands of hydrogen bonding shifted from 3287 cm^−1^ to 3315 cm^−1^, indicating that strong hydrogen bonds might formed between gum Arabic and fucoxanthin and black seed oil. Moreover, the eliminations of some bands revealed the embedding and encapsulation of fucoxanthin [48]. 

According to the FTIR spectrum of the casein, the band at 1643 cm^−1^ was characterized as amide I and was attributed to stretching vibration of C=O in –CONH groups. The amide II band arising from NH bending vibration and C-N stretching vibration was distributed at 1537 cm^−1^. The appearance of amide I and II α-helical confirmed the configuration of casein [49,50]. In the FTIR spectrum of casein-stabilized nanoemulsion, some peaks around 700–900 cm^−1^ were disappeared, and the band at 1537 cm^−1^ slightly shifted to 1635 cm^−1^ and 1643 cm^−1^ shifted to 1743 cm^−1^, which was related to proteins [51]. These observations indicated the interaction between protein and fucoxanthin and were in agreement with Li et al. [51].

### 2.6. TEM

**Figure 4:**
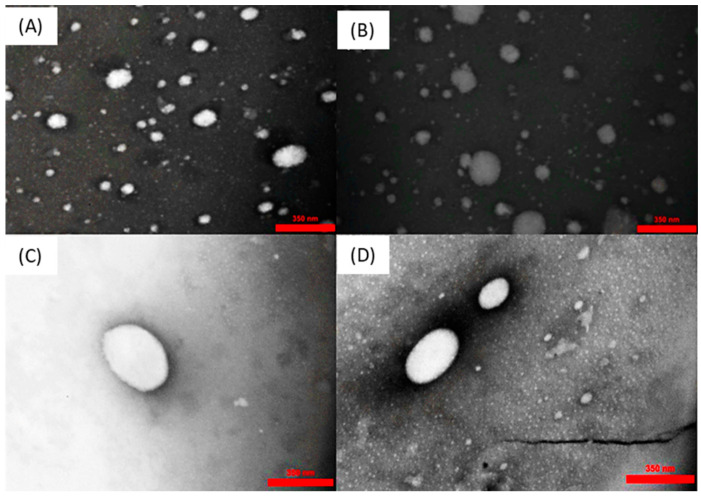
TEM images of nanoemulsion stabilized with 2% tween 80 (**A**), sodium caseinate (**B**), gum Arabic (**C**), and fucoidan (**D**) after 15 days.

The morphology of droplets is an important feature in the emulsions. The morphological characterization of nanoemulsions prepared with 2% emulsifiers after 15 days was visualized by TEM, as shown in Figure 4. Nanoemulsions had a spherical shape with an average droplet size of less than 300 nm. The TEM micrographs confirmed the results obtained from droplet size analysis using the DLS.

### 2.7. In Vitro Release

**Figure 5:**
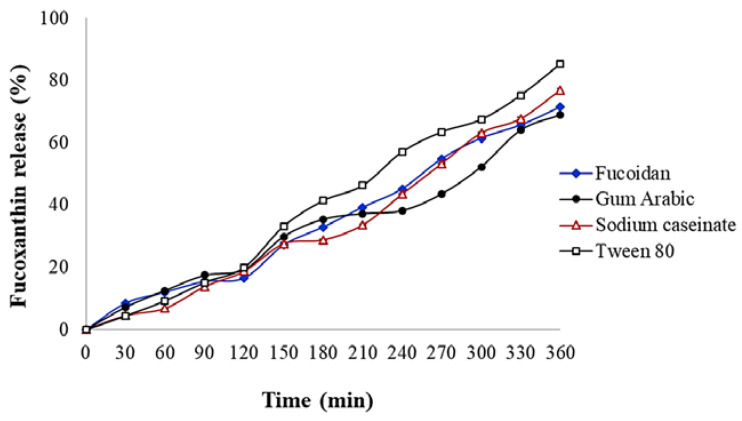
Fucoxanthin release from different nanoemulsions stabilized with 2% emulsifiers.

Fucoxanthin metabolism is accompanied by hydrolysis to fucoxanthinol by digestive enzymes, lipase, and cholesterol esterase in the gastrointestinal tract and then converted to amarociaxanthin A in the liver [52]. To investigate the in vitro release of fucoxanthin, the samples prepared with 2% emulsifiers were chosen to be submitted to gastrointestinal conditions. Figure 5 exhibits the fucoxanthin release from nanoemulsions in SGF and SIF. The lowest release rate of fucoxanthin was observed in gum Arabic (*p* < 0.05), followed by fucoidan < sodium caseinate < tween 80. The controlled release of encapsulating materials depends on their droplet size and structural properties. During the simulated gastrointestinal in vitro digestion, a higher amount of fucoxanthin release was observed in the tween 80-stabilized emulsion (85.14 ± 0.16%). Among natural emulsifiers, the fucoxanthin release reached a value close to 76.91 ± 0.34% in the sodium caseinate-emulsified sample, which indicated that the fucoxanthin release was influenced by the particle size as the smaller droplet size prepared a larger surface area available for enzymes. In addition, tween 80 was more stable against SGF conditions and aggregation compared with natural emulsifiers because the acidic pH of the stomach phase caused a reduction of ionic strength and electrostatic repulsion between the oil droplets, resulting in aggregation and coalescence [13]. It seems that tween 80 is not influenced by the acidic conditions. Moreover, the higher fucoxanthin release in SIF compared with SGF indicated the higher stability of nanoemulsions against the acidic conditions. It seems that the initial release of fucoxanthin was influenced by emulsifier type. When the sodium caseinate-prepared nanoemulsions were exposed to the SGF, the flocculation of casein under isoelectric pH slowed down the release of fucoxanthin (about 27%). The presence of these large, dense, aggregated casein emulsifiers may inhibit the complete digestion of fucoxanthin at the end of SIF [13]. The burst release of fucoxanthin when exposed to SIF was observed, which might be related to the relative weakening of interfacial layers in contact with the SGF fluid. Generally, fucoxanthin release from the polysaccharides-based emulsions occurred relatively rapidly at the initial stage of digestion and then more gradually at later times. It seems that the structural attributes were more effective than droplet size. During the initial 120 min, the fast release rate of fucoxanthin from polysaccharides-based nanoemulsions could be due to the maintaining of oil droplets weakly together by a larger structure of polymers. These phenomena caused the rapid release of unencapsulated fucoxanthin when exposed to SGF. While fucoxanthin trapped in the nanoemulsion was slowly released at the further stages. Although, the fucoidan- and gum Arabic-stabilized nanoemulsions had a similar digestion profile, the fucoxanthin release from fucoidan-based nanoemulsion was higher in SGF, which might be related to the fucoidan open structure, which makes it penetrable to water and enzymes [13]. In addition, the gum Arabic has a relatively low affinity for oil and water and was replaced easily from droplet surfaces which causes coalescence, lower available sites for enzymes, and lower release [16]. Our results were in agreement with the β-carotene bioaccessibility from the emulsion stabilized by gum Arabic [32] and the curcumin in vitro release from zein-fucoidan nanoparticles [47].

In addition, there are some other pathways for absorption of the nanoparticles in the gastrointestinal tract (GT) and those targeting M cells. The digestion and release rate of the encapsulated bioactive components are also influenced by particle size and surface charge of polymers. The mucus layer of the small intestine is masked by negatively charged mucin glycosides. Thus, positively charged polymers tend to adhere to the negative charge of the mucus layer. Furthermore, proteins with negative charges can interact with glycoproteins. Glycoproteins act as unique compounds for the absorption of proteins by M cells [53]. Moreover, during the GT, destabilization of droplets may occur by alternating the electrostatic interactions due to changes in the pH and ionic strength [14]. The larger oil droplets also appear in emulsion stabilized by proteins in high ionic strength or acidic conditions indicating the occurrence of droplet coalescence and therefore a decrease in droplet surface area. In GT, protease breaks down the structure of a protein in the emulsion, which contributes to the destabilization of emulsion and direct lipase–oil contact facilitates faster lipolysis [14,54]. The digestion of proteins in the GT begins firstly by gastric protease, and about 18% of casein is digested in SGF for 2 h [55]. Moreover, Huang et al. [9] reported that the release of fucoxanthin and lipolysis depends on the droplet size of the emulsion. As the smaller size of droplets means a larger surface area, when exposed to the digestive enzymes, they induced the rapid release [9]. In the case of polysaccharides, Saravana et al. [35] reported that the nanoemulsion stabilized by k-carrageenan was susceptible to aggregation, and the increase in droplet size occurred during bypassing the gastric phase and exposure to SGF. During the further digestion, the carrageenan molecules adsorbed on the surface of the oil droplets were hydrolyzed, resulting in fucoxanthin release and higher bioavailability [35].

In addition, the kinetic parameters obtained are listed in Table 3. Release–kinetic correlation coefficients (R2) of nanoemulsions were greater than 0.95. The diffusional exponent (*n*) varied between 0.497 and 0.508. These results indicate that the fucoxanthin release follows a Fickian diffusion, where the release is governed by diffusional behavior. 

## 3. Materials and Methods

### 3.1. Extraction of Fucoxanthin

Extraction of fucoxanthin was carried out by our previous study [1]. The powdered *S. angustifolium* was extracted three times with 90% ethanol (1:20 *w/v*) and filtered. Then the filtrate was concentrated by rotary evaporator and loaded to column chromatography with silica gel (particle size 100–200 mesh) for purification. Elution was performed with n-hexan-aceton (6:4; *v/v*) as a mobile phase to recover fucoxanthin. Finally, the residual fucoxanthin was eluted by acetone. All of the extract solutions were together concentrated by rotary evaporation. Purification analysis of fucoxanthin was performed with HPLC system (KNAUER, Berlin, Germany) equipment with UV/Vis detector 2600 and C18 column (sphere-image, ODS-2, 300 × 4 mm; 5 µm) and methanol/acetonitrile (50:50 *v/v*) as the mobile phase, which was eluted in the column at a 0.6 mL/min flow rate. Fucoxanthin was detected at 450 nm, and the results were compared to the fucoxanthin standard. The recovered fucoxanthin exhibited purity of about 54% on HPLC.

### 3.2. Extraction of Fucoidan

Fucoidan extraction was performed in accordance with Hifney, Fawzy [56] with some modifications. The remaining seaweed powder from the fucoxanthin extraction process was heated with distilled water (1:20; *w/v*) at 65–70 °C for 3 h under acidic condition (pH ≃ 3 with 0.1 M HCl). After extraction, the supernatant was separated by centrifugation (6000 rpm for 10 min) at room temperature. Then, 1% CaCl_2_ was added and stored at 4 °C overnight to precipitated alginate present. The resulting precipitate was removed by centrifugation, and the supernatant was treated with 2 volumes of ethanol (96%), and it was left to precipitate fucoidan for 12 h at 4 °C. Finally, the extracted fucoidan was freeze-dried (Christ, Osterode am Harz, Germany).

### 3.3. Nanoemulsions Preparation

Three different concentrations of natural emulsifiers including fucoidan, gum Arabic, and sodium caseinate (0.5%, 1% and 2% wt) were used in the aqueous phase of O/W emulsion. The nanoemulsion was formulated using 10% wt black seed oil and 90% aqueous phase. The oil phase of the emulsion was prepared by mixing black seed oil with 400 ppm fucoxanthin using magnetic stirring for 30 min. The aqueous phase was prepared by homogenizing the mixture of emulsifier and water with ultra-turrax T25 homogenizer (IKA, Staufen, Germany), followed by the dropwise addition of the oil phase for 5 min at 12,000 rpm. Then, the emulsion was subjected to ultrasonic treatment (150 W, amplitude 80%, Ultrasonic Homogenizer, SONOPULS HD3200, Bandelin, Berlin, Germany) for 10 min. To avoid temperature effects, the tube with emulsion prepared by homogenization was placed into a beaker with ice. Similar preparation was performed using tween 80 as an emulsifier [57].

### 3.4. Droplet Size and Zeta-Potential Measurement

Dynamic light scattering (DLS) is the most common approach to analyze hydrodynamic particle size and distribution of the particles over a range of sizes. The average droplet size, polydispersity index (PI), and zeta potential of the fresh nanoemulsions were determined by dynamic light scattering (DLS, SZ 100, Horiba, Kyoto, Japan). Samples were properly diluted with distilled water (1:100) to avoid multiple scattering effects. About 1 mL of diluted sample was added to a polystyrene latex cell and the measurement was carried out at 25 °C.

### 3.5. Emulsion Stability and Creaming Index

Emulsion stability was estimated using the method based on the creaming index. In the test tube, about 5 mL of the freshly prepared emulsion was poured and stored at room temperature for 15 days. Emulsion stability was calculated by the ratio between the height of cream (H) and the initial height of the emulsion (H_0_). The best samples were used for further analysis [38].

### 3.6. Encapsulation Efficiency (EE)

EE was determined according to the method described by Richa and Choudhury [57] with slight modifications. In total, 2 mL of samples were placed inside a Dialysis tubing membrane (MW cut-off ~12–14 kD, pore size 2.4 nm,) and immerged in 10 mL of ethanol for 2 h at room temperature. Then, the mixture was centrifuged at 5000× *g* for 10 min. The amount of fucoxanthin found in the supernatant was evaluated by UV-Vis spectrometry, at 450 nm. The EE % was obtained according to Equation (1).

(1) $$\mathrm{EE}\%=\frac{\mathrm{total}\;\mathrm{amount}\;\mathrm{of}\;\mathrm{fucoxanthin}\;-\;\mathrm{free}\;\mathrm{fucoxmathin}\;}{\mathrm{total}\;\mathrm{amount}\;\mathrm{of}\;\mathrm{fucoxanthin}}\;\times \;100$$

### 3.7. Fourier-Transform Infrared (FT-IR) Spectroscopy

The FT-IR spectrum of samples was obtained by FTIR spectrophotometer (Tensor II, Bruker, Germany). A small drop of prepared emulsions was placed on the KBr plate, and then, we placed a second plate on the sample in order to spread the liquid in a thin layer between plates. Spectra were taken in the wavenumber range of 4500–500 cm^−1^.

### 3.8. Transmission Electron Microscopy Analysis

Transmission electron microscopy (TEM) analysis was used to image the morphology of fucoxanthin nanoemulsions after 15 days. A single droplet of diluted emulsions (1:300) was placed on a copper carbon=coated grid and then dyed by uranyl acetate. A piece of filter paper was used to wipe off the excess stain from the grid and was left at ambient temperature to dry. Images were analyzed on a TEM (LEO-906E, Carl Zeiss, Jena, Germany) at an accelerating voltage of 80 kV.

### 3.9. In Vitro Release

The in vitro release of fucoxanthin was measured according to the method described by Oliyaei, Moosavi-Nasab [1] with some modifications. Fucoxanthin release from nanoemulsion was examined in simulated gastric fluid (SGF, pH 1.2) containing 0.3% *w/v* pepsin and simulated intestinal fluid (SIF, pH 7.5) containing 0.1% *w/v* pancreatin and lipase (0.2% *w/v*) at 37 °C. Next, 2 mL of each sample was placed in a dialysis tubing membrane (MW cut-off ~12–14 kD) containing 10 mL of SGF and shaken for 120 min. Then, the dialysis tube was transferred to SIF for 240 min. Sampling was performed each 30 min after centrifugation at 5000×*g* for 5 min. The supernatants were collected for fucoxanthin release assay and were determined based on absorbance at 450 nm and according to the Equation (2):

(2) $$\mathrm{Fucoxanthin}\;\mathrm{release}\;\left(\%\right)=\frac{M_{0}-M_{t}}{M_{0}}\;\times 100$$

where M_0_ is the fucoxanthin initially encapsulated and M_t_ is the fucoxanthin remaining in the emulsion at a given incubation time.

Moreover, to predict the release behavior of fucoxanthin from emulsions, the release data fitted to a well-known Korsmeyer–Peppas model [58]:

$$M_{t}/M_{∽}=k.t^{n}$$

where M_t_ /M_∽_ is a fraction of fucoxanthin release at time t, k is the characteristics of the carrier network system, and n is the release exponent indicative of the mechanism of release. Values for n lower than 0.5 indicate a Fickian diffusion mechanism. Values higher than 0.5 correspond to a non-Fickian release.

### 3.10. Statistical Analysis

Data were reported as the mean standard deviation for triplicated determinations that were analyzed using analysis of variance (ANOVA) and Duncan’s multiple range test at *p* < 0.05 by SAS software (SAS Institute, Cary, NC, USA).

## 4. Conclusions

In summary, three types of natural emulsifiers (fucoidan, gum Arabic, and sodium caseinate) were used for fucoxanthin encapsulation and compared with tween 80. The best encapsulation efficacy was obtained from the tween 80-stabilized nanoemulsion, followed by sodium caseinate, fucoidan, and gum Arabic nanoemulsions. Among the natural emulsifiers, the smaller droplet size was observed in sodium caseinate-emulsified nanoemulsion, which should be responsible for higher stability and encapsulation efficacy. The release rate of fucoxanthin from natural nanoemulsions was directly correlated with the types of emulsifiers and droplet size. All natural emulsifiers presented different in vitro releases, and a higher fucoxanthin release was observed in sodium caseinate-stabilized nanoemulsion. The protein-based emulsifier showed better function; however, fucoidan and gum Arabic exhibited a relatively high encapsulation efficacy and fucoxanthin release. For future trends, it is suggested to use fucoidan in combination with other natural emulsifiers or with a low amount of tween 80 to improve the stability of nanoemulsions. Additionally, a better understanding of the influence of algae species and the chemical structure of fucoidan on its functional and biological properties is needed. So far, most research on fucoidan have been versatile and promising in many pharmaceutical aspects, not only as an emulsifier, but also as wall material for encapsulation or its biological attributes. As fucoidan, fucoxanthin, and black seed oil have several biological attributes, the development of therapeutic nanocarriers are essential. In addition, in vivo studies of therapeutic effect of fucoidan-based nanoemulsions on chronic disorders are suggested. These investigations open opportunities to develop advanced nanoemulsions as prospective nanocarriers with a wide range of applications.

## Figures and Tables

**Table 1:** Average particle size and PDI of different nanoemulsions.

Emulsifiers	Initial Average Particle Size (nm)	PDI
Fucoidan (1%)	143.67 ± 3.35 ^Bb^	0.31 ± 0.01 ^Bab^
Gum Arabic (1%)	148.00 ± 4.75 ^Bb^	0.42 ± 0.10 ^Aa^
Sodium caseinate (1%)	161.80 ± 1.95 ^Ba^	0.41 ± 0.04 ^Aab^
Tween 80 (1%)	128.53 ± 1.80 ^Bc^	0.27 ± 0.11 ^Ab^
Fucoidan (2%)	126.73 ± 2.68 ^Bc^	0.36 ± 0.03 ^Aa^
Gum Arabic (2%)	127.50 ± 2.62 ^Bc^	0.34 ± 0.08 ^Aa^
Sodium caseinate (2%)	113.43 ± 1.29 ^Bd^	0.33 ± 0.06 ^Aa^
Tween 80 (2%)	113.27 ± 0.45 ^Bd^	0.29 ± 0.03 ^Aa^

Data are expressed as mean ± SD (*n* = 3). Different small letters represent significant differences between different samples in each level, and capital letters represent significant differences indifferent levels in each sample (*p* < 0.05).

**Table 2:** Encapsulation efficiency of fucoxanthin-loaded nanoemulsions’ encapsulation efficiency (%).

Emulsifiers	Encapsulation Efficiency (%)
Fucoidan (1%)	72.57 ± 0.11 ^Bc^
Sodium caseinate (1%)	85.01 ± 0.09 ^Bb^
Gum Arabic (1%)	59.34 ± 0.12 ^Bd^
Tween 80 (1%)	89.22 ± 0.16 ^Ba^
Fucoidan (2%)	79.32 ± 0.09 ^Ac^
Sodium caseinate (2%)	88.51± 0.11 ^Ab^
Gum Arabic (2%)	60.34 ± 0.13 ^Ad^
Tween 80 (2%)	92.15 ± 0.15 ^Aa^

Data are expressed as mean ± SD (*n* = 3). Different small letters represent significant differences between different samples in each treatment and capital letters represent significant differences in different levels in each sample (*p* < 0.05).

**Table 3:** Kinetic parameters obtained from release curve for fucoxanthin-loaded nanoemulsions.

Nanoemulsions	Kinetic Parameters		
	*n*	k	R^2^
Fucoidan	0.498	0.201	0.988
Gum Arabic	0.508	0.179	0.977
Sodium caseinate	0.499	0.215	0.980
Tween 80	0.497	0.243	0.992

## Data Availability

Not applicable.
